# Visualizing Empathy in Patient-Practitioner Interactions Using Eye-Tracking Technology: Proof-of-Concept Study

**DOI:** 10.2196/57884

**Published:** 2024-12-11

**Authors:** Yuyi Park, Hyungsin ­Kim, Hakkyun Kim

**Affiliations:** 1 Department of Dentistry, Seoul National University, Seoul, Republic of Korea; 2 Graduate School of Techno Design, Kookmin University, Seoul, Republic of Korea; 3 School of Business, Sungkyunkwan University, 25-2 Sungkyunkwan-ro, Jongno-gu, Seoul, 03063, Republic of Korea, 82 822-760-0479

**Keywords:** clinical empathy, eye tracking, medical communication, nonverbal behavior, doctor-patient encounters

## Abstract

**Background:**

Communication between medical practitioners and patients in health care settings is essential for positive patient health outcomes. Nonetheless, researchers have paid scant attention to the significance of clinical empathy in these interactions as a practical skill.

**Objective:**

This study aims to understand clinical empathy during practitioner-patient encounters by examining practitioners’ and patients’ verbal and nonverbal behaviors. Using eye-tracking techniques, we focused on the relationship between traditionally assessed clinical empathy and practitioners’ actual gaze behavior.

**Methods:**

We used mixed methods to understand clinical encounters by comparing 3 quantitative measures: eye-tracking data, scores from the Korean version of the Jefferson Scale of Empathy–Health Professional, and Consultation and Relational Empathy survey scores. We also conducted qualitative interviews with patients regarding their encounters.

**Results:**

One practitioner and 6 patients were involved in the experiment. Perceived empathy on the part of the practitioner was notably higher when the practitioner focused on a patient’s mouth area during the consultation, as indicated by gaze patterns that focused on a patient’s face. Furthermore, an analysis of areas of interest revealed different patterns in interactions with new as opposed to returning patients. Postconsultation interviews suggested that task-oriented and socially oriented empathy are critical in aligning with patients’ expectations of empathetic communication.

**Conclusions:**

This proof-of-concept study advocates a multidimensional approach to clinical empathy, revealing that a combination of verbal and nonverbal behaviors significantly reinforces perceived empathy from health care workers. This evolved paradigm of empathy underscores the profound consequences for medical education and the quality of health care delivery.

## Introduction

Communication between practitioners and their patients is crucial in medical services as the first entry point into the health care system [1]. In particular, communication in health care settings is gaining more attention as the new paradigm of global health shifts toward patient-centered care [2]. Practitioner-patient communication in health care settings is necessary for establishing a trusting relationship and is a fundamental aspect of improving diagnostic accuracy and delivering effective clinical care [1]. In addition, research shows that practitioners’ empathy abilities, when used in health care processes, enhance the quality of health care and patients’ health outcomes [3].

Clinical empathy is a concept that includes several competencies of health care professionals, namely, the ability to understand a patient’s experiences and perspectives, the capacity to respond appropriately to the patient’s emotions, and the skills to communicate effectively [4-6]. As in everyday communication, health communication involves the appropriate use of verbal and nonverbal communication skills [7]. Research estimates that 80% of communication is nonverbal, and the nonverbal communication of health care professionals is crucial in influencing patients’ well-being and their overall care experience [8,9]. In addition, nonverbal components may be more important than verbal components, especially when clinicians make clinical decisions involving multicultural patients [10].

Despite a gradual growth in research on health communication and clinical empathy, to date, relatively few studies have analyzed the nonverbal communication aspects of medical consultations. A review of previous studies revealed that most of them were not systematic in their investigation of self-reported perceptions of or attitudes toward clinical empathy among patients [11,12], practitioners [13,14], and students [15,16]. These survey-based studies (particularly those solely reliant on self-report questionnaires) are susceptible to bias in terms of reporting or social desirability and, therefore, are limited in their interpretation of the genuine characteristics of empathetic interactions [17].

To address these issues and obtain more objective data, researchers have increasingly applied eye-tracking technology in various fields, including medicine. Eye-tracking technology provides valuable information about an individual’s attention, perception, memory, cognitive workload, and cognitive processes, and it is noninvasive [18,19]. Consequently, this technology has been used to analyze the gaze behavior of health care workers in real-world settings, leading to improvements in educational and clinical training processes, treatment and diagnostic outcomes, and the medical environment [20-23]. In addition, studies have been conducted to explore patient gaze behavior to enhance patients’ experience [24-26]. However, there is a paucity of studies that apply eye-tracking technology to real-life interactions between health care workers and patients.

Therefore, in this study, we explore gaze behaviors as a key nonverbal component of social interaction in the context of clinical empathy. This study used an eye-tracking device worn by a practitioner during medical consultations to understand eye movements that were characteristic of empathy.

## Methods

### Design

In this study, we used an exploratory research design to investigate the use of eye-tracking glasses in practitioner-patient encounters. We also incorporated mixed methods to collect data to understand practitioner-patient interactions. In addition, we collected qualitative data from patients at the end of the experiment through interviews. Ultimately, we triangulated the data to cross-verify and validate the findings from the eye-tracking technology with traditionally measured empathy assessments, such as the Jefferson Scale of Empathy–Health Professional (JSE-HP) and the Consultation and Relational Empathy (CARE) survey.

### Setting

We conducted our experiment from December 2021 to February 2022 at the office of a practitioner of traditional Korean medicine in Incheon, South Korea. We used eye-tracking technology throughout the experiment; however, we only considered individual patients’ medical consultation sessions for analysis. The start and end points of the consultation were the initial greeting and the closing exchange between the patient and the practitioner. To avoid potential distractions for the practitioner and prevent the recording of patients’ personal information through the eye-tracker, we opted to use paper charts rather than an electronic medical record system. In this experiment, the practitioner documented only the minimum information necessary (vital signs, practitioner’s findings, and prescriptions) on the paper chart.

### Participants

We used social media platforms to disseminate recruitment flyers to enroll a practitioner participant. Given that the study was conducted at the practitioner’s office, recruitment flyers for patient participants were displayed on the office’s bulletin board. Ultimately, 7 participants were recruited for the study, which included 1 practitioner and 6 patients. The practitioner, a woman in her mid-30s, specialized in traditional Korean medicine and ran her own clinic. The patient group comprised 1 male and 5 female patients, spanning a diverse age range from their 20s to their 60s. The study excluded individuals taking neuropsychiatric medications, practitioners who wore glasses, and psychiatrists. These exclusions aligned with our research objective, which was to understand clinical empathy comprehensively within general medical interactions. The experiment was conducted during the COVID-19 pandemic, when wearing masks was legally mandated in hospitals in South Korea. To ensure that the participants were safe and that their lips were visible, they wore transparent masks.

### Materials

We used the Tobii Pro Glasses 2.0 (Tobii AB) to track eye movements. This wireless wearable eye-tracking device records the position of a subject’s pupil with infrared cameras and maps the subject’s visual attention. It weighs 45 grams, so a user can move freely while wearing the glasses. We analyzed all the recorded videos using the Tobii Pro Lab Analyzer software. To visualize and quantify the eye-tracking data, we used two methods: (1) heat maps, which display the distribution and frequency of the practitioner’s gaze as an image, and (2) metrics derived from areas of interest (AOIs), which measure the practitioner’s attention to specific areas by fixation duration analysis. Then, we used 2 standardized instruments to measure clinical empathy, namely, the Korean version of the JSE-HP [27] for the practitioner and the CARE survey [11] for the patients. Finally, we developed a semistructured interview questionnaire to gain insights into the patients’ perceived empathy with the practitioner. We used iterative processes based on inductive content analysis to ensure the reliability of the qualitative findings. The main findings were shared with 2 patient participants, and their feedback was then incorporated. Microsoft Word and Excel were used for coding and analysis.

### Procedures

The experiment consisted of 3 phases: a pre–medical consultation phase, a medical consultation phase, and a post–medical consultation phase. In the initial phase, the practitioner and all 6 patients completed a consent form. Additionally, the practitioner took the Korean JSE-HP assessment once to evaluate her clinical empathy capabilities. Following this assessment, she prepared for the experiment by putting on the Tobii eye-tracking glasses in preparation for the medical consultation phase. During this phase, the practitioner engaged with each of the 6 patients individually while wearing eye-tracking glasses. In the post–medical consultation phase, the practitioner took off the eye-tracking glasses and transcribed the notes recorded on paper during the consultation into the electronic medical record system. After the consultation, the patients moved to a separate area outside the consulting room and assessed the level of clinical empathy they had experienced using the CARE tool. Lastly, we conducted a 15-minute semistructured interview with each patient to explore wider aspects of clinical empathy.

### Ethical Considerations

All of the procedures were reviewed and approved by the Kookmin University Institutional Review Board (KMU-202111-HR-289). The participants, including both the practitioner and the patients, were given ample time to review the study information sheet, which outlined the research purpose, procedures, data analysis methods, participants’ rights, and other relevant information. Informed written consent was obtained from all participants before they participated in the medical consultations. Each participant was offered a nominal compensation of 70,000 KRW (US $50) for their time and participation. To ensure privacy and confidentiality, all of the data collected were anonymized by assigning unique participant identification numbers at the point of data collection.

## Results

### Practitioner’s Self-Reported Empathy

The practitioner scored 94 out of a maximum of 126 points on the Korean JSE-HP. This self-report scale consists of 18 questions, each rated on a 7-point Likert scale. In a departure from the original JSE-HP scale, the Korean edition omits 2 questions for better internal consistency. The Korean edition of the JSE-HP assesses clinical empathy across 3 subscales: 10 items on perspective taking, 6 items on compassionate care, and 2 items on standing in the patient’s shoes. Higher scores indicate greater proficiency in achieving clinical empathy [4,27]. The practitioner’s score (94 points) was similar to previously reported scores in a study by Park et al [28], which assessed empathy among Korean medical residents using the same scale (male participants had a score of 92.4 and female participants a score of 95.8).

### Patients’ Perceptions of the Practitioner’s Empathy

The average score for the Korean version of the CARE tool, which was designed for patients to evaluate their practitioner’s empathy, was 47.8 out of a possible 50 points. Generally, a score above 43 points implies a high level of empathy in practitioners [29]. Since this practitioner’s score was almost perfect (with a maximum of 50 points), her empathy ability was judged to be very high. The Korean CARE instrument consists of 10 validated and reliable items rated on a 5-point Likert scale. A higher total score indicates increased perceptions of clinical empathy by patients. Table 1 gives an overview of the demographic characteristics of each participant in the study, including their respective Korean CARE scores. Additionally, we asked participants about their status as either new or follow-up patients.

**Table 1.** General characteristics and empathy ratings of the patient participants.

Subject ID	Type of visit	Age (years)	Gender	Chief concern	CARE^a^ score (Korean edition; maximum score 50)
1	Follow up	21	Female	Gastrointestinal discomfort	50
2	Follow up	21	Female	Lumbar and leg pain	48
3	First time	63	Female	Lumbar, right knee, and leg discomfort	39
4	Follow up	45	Female	Neck and shoulder pain	50
5	First time	38	Female	Head, neck, arm, shoulder, and hand pain	50
6	Follow up	20	Male	Neck, shoulder, and lumbar pain	50

a CARE: Consultation and Relational Empathy Measure.

### AOIs During the Medical Consultation

To understand where the practitioner focused her attention during the medical consultation, we defined 4 AOIs. These AOIs included only the critical components of the consultation: (1) the patient’s face, (2) the patient’s body, (3) the patient’s paper chart and the practitioner’s office environment, and (4) educational materials provided for the patient. Gazes that fell outside these areas were categorized as “white space.” In addition, due to the varying length of the consultations, we quantified the practitioner’s gaze time for each area as a percentage of the total time spent, as shown in Table 2.

The analysis revealed that for all 6 patients, the longest gaze time was directed toward the patients’ faces. However, when examining the groups’ results separately, differences in AOI distributions became apparent. In the follow-up patient group, the average AOI distribution was highest for the face (mean 32.4%, SD 14.4%), followed by the chart and environment (mean 10.4%, SD 6.7%), the body (mean 7.7%, SD 8%), and educational materials (mean 6.9%, SD 8.9%). Similarly, in the first-time patient group, the face received the most attention (mean 28.4%, SD 5.3%), but the subsequent proportions differed: educational materials (mean 14.3%, SD 4.9%), the chart and environment (mean 7.4%, SD 1.1%), and the body (mean 7%, SD 0.6%). This indicates that the practitioner allocated comparable attention to patients’ faces, bodies, and the chart and environment for both the first-time and returning patients. Additionally, we observed a notable disparity in attention given to patient educational/explanatory materials, with a more than 2-fold difference—a mean 14.3% (SD 4.9%) for first-time patients compared to a mean of only 6.9% (SD 8.9%) for follow-up patients.

**Table 2.** Practitioner’s eye-tracking data for different areas of interest (AOIs). Percentages are calculated with the value in each row’s entry in the “Length of medical consultation” column as the denominator.

Type of visit		Length of medical consultation (seconds)	Time spent in each AOI (seconds), n (%)				
			Face	Body	Chart and environment	Educational material	White space^a^
**Follow up**							
	Subject ID 1	243.7	124.2 (51)	2.2 (0.9)	46.7 (19.2)	0.0 (0)	70.6 (29)
	Subject ID 2	148.9	53.9 (36.2)	2.9 (2)	17.6 (11.8)	0.0 (0)	74.4 (50)
	Subject ID 4	157.3	29.3 (18.6)	28.6 (18.2)	6.6 (4.2)	29.4 (18.7)	63.3 (40.2)
	Subject ID 6	254.8	60.6 (23.8)	24.1 (9.5)	16.1 (6.3)	22.5 (8.8)	131.4 (51.6)
	Mean	—^b^	67.0 (32.4)	14.5 (7.7)	21.8 (10.4)	13.0 (6.9)	84.9 (42.7)
**First time**							
	Subject ID 3	565.3	181.6 (32.1)	42.1 (7.4)	37.3 (6.6)	61.2 (10.8)	243.1 (43)
	Subject ID 5	271.5	66.7 (24.6)	17.9 (6.6)	22.3 (8.2)	48.1 (17.7)	116.4 (42.9)
	Mean	—	124.2 (28.4)	30.0 (7)	29.8 (7.4)	54.7 (14.3)	179.8 (43)

a White space indicates any areas outside the defined AOI.

b Not applicable.

### Heatmap During the Medical Consultation

**Figure 1.**
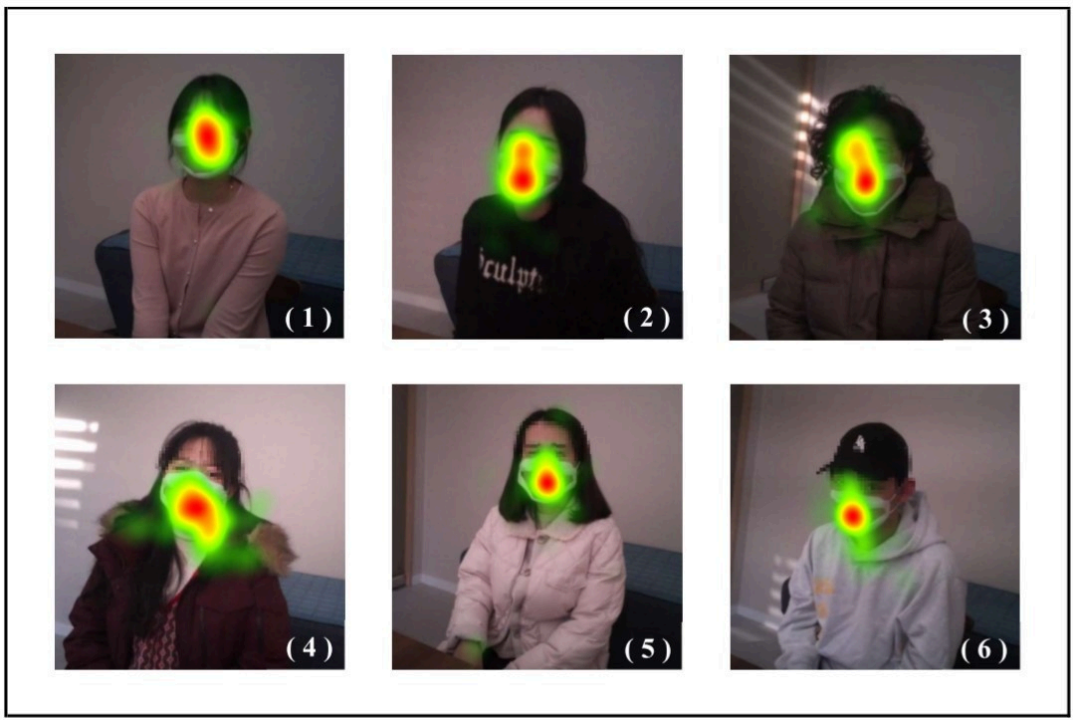
Heat maps of the patients’ images. Red indicates areas of highest visual attention, while yellow/orange indicates areas of moderate focus. Green represents areas with relatively low gaze activity, and colorless areas correspond to areas that received little to no attention. Numbers represent patient number.

We used the practitioner’s gaze (fixation) data to create a heat map for each of the 6 patients (Figure 1). These maps show the distribution of the practitioner’s gaze fixation position toward the patient. Warmer colors generally indicate higher attention, while cooler colors represent lower attention. Our data show red for high gaze intensity, yellow for moderate gaze intensity, and green for low gaze intensity. During the medical consultations, most of the practitioner’s gaze distribution was a long ellipse centered on the patient’s mouth (participants 1, 2, 3, 5, and 6). Notably, the area around the mouth emerged as the area of strongest visual attention in all patients, except for patient 1. Moreover, the practitioner’s visual attention occasionally extended to areas where patients reported discomfort (patient 4: neck and shoulders; patient 5: head and hand), though this pattern was most pronounced in patient 4.

### Post–Medical Consultation Interview

Our analysis of the interview data concentrated on identifying the verbal and nonverbal behaviors of practitioners that may enhance patients’ perceptions of empathy during medical consultations. As detailed in Table 3, we discerned 12 distinct themes, organized into 4 subcategories, and further consolidated into 2 overarching categories, representing the patients’ perceptions of practitioners’ clinical empathy while distinguishing between task-oriented and socially oriented behaviors.

First, the task-oriented category included clinical empathy rooted in essential or functional medical tasks within the health care environment. Patients perceived the presence of task-oriented clinical empathy when a practitioner exhibited commitment to listening attentively to the patient; demonstrated a sincere commitment to addressing the patient’s holistic needs, beyond the completion of medical tasks; and fostered an empathetic environment by delivering thorough, in-depth explanations tailored to the patient’s comprehension level. Conversely, a lack of task-oriented clinical empathy became apparent in patients’ perceptions when a practitioner listened inattentively, delivered care that fell short of the patient’s expectations, and demonstrated insufficient verbal communication during practitioner-patient interactions. For example, the participants mentioned the following:

I believe that when doctors listen actively to patients and truly understand them, it is a way to demonstrate empathy.

[Participant 2]

I felt a sense of clinical empathy from my doctor when she understood the severity of my pain and took proactive measures in my treatment.

[Participant 3]

During the medical consultation, I discussed my pain with the doctor, but it felt like she was not really listening. She spoke as if she already knew everything. I realized that she just wanted to wrap up the consultation, so I didn’t feel any empathy.

[Participant 4]

Second, the socially oriented category extended beyond the obligatory medical tasks conducted by practitioners in health care settings, including expressions of care and concern for the patient and providing comfort. Interviewees expressed perceptions of socially oriented clinical empathy when a practitioner considered broader aspects of the patient’s health and life beyond their immediate medical issue, when the interaction respected the patient, when the practitioner conveyed positive attitudes and words of encouragement regarding the patient’s recovery, and when the practitioner actively sought to understand the patient’s perspective and feelings about their situation. In contrast, in interactions lacking socially oriented clinical empathy, the practitioner manifested inappropriate nonverbal communication or unkind and rude behavior toward the patient. For example, patients said the following:

Doctors’ friendly attitude and commitment to patient care are crucial in clinical empathy.

[Participant 1]

When my doctor noticed that I had to go to work even though I was sick, he showed concern for me and offered encouraging words about my recovery; it made me feel that he empathized with my situation.

[Participant 5]

I shared my new symptoms with my doctor. However, I didn’t perceive any moments of empathy because the doctor focused solely on the computer monitor and prescribed medication without asking any questions.

[Participant 6]

**Table 3.** Factors associated with patients’ perceptions of clinical empathy.

Category, subcategory, and theme		
**Task-oriented clinical empathy**		
	**Presence of task-oriented clinical empathy**	
		Listening attentively Offering sincere or active care Supplying in-depth explanations
	**Lack of task-oriented clinical empathy**	
Listening inattentively Delivering low-quality care Inadequate verbal communication
**Socially oriented clinical empathy**		
	**Presence of socially oriented clinical empathy**	
		Showing concern for the patient’s well-being Displaying a kind and polite demeanor Providing reassurance Understanding the patient’s perspective or emotions
	**Lack of socially oriented clinical empathy**	
		Inappropriate nonverbal communication Impolite manners

## Discussion

### Principal Findings

This study used eye-tracking technology to capture and analyze the visual attention patterns of a practitioner during real-world medical consultations. We analyzed the data alongside the practitioner’s self-reported empathy scores and the empathy scores assigned to the practitioner by her patients. The analysis revealed that the patients rated the practitioner’s empathy highly and also that the practitioner focused predominantly on the patients’ mouth area during consultations. Furthermore, postconsultation interviews demonstrated that patients considered both verbal and nonverbal aspects of empathy to be important in clinical interactions. From these results, we derived 3 interesting implications.

First, the analysis showed that the practitioner demonstrated a high level of clinical empathy by tending to focus her gaze more on the facial areas of the patients during the consultations. This finding aligns with the outcomes of various previous studies [30-32]. Scholars have identified the additional gaze frequency or time that practitioners spend observing patients’ faces as a crucial element in improving patient satisfaction and the overall quality of practitioner-patient interactions [33,34].

Second, our observations of facial interactions highlighted a distinct emphasis on the patients’ mouths. The heatmap analysis indicated that the practitioner, who demonstrated a high level of clinical empathy, had a vertically elongated oval pattern of gaze centering on the mouth. This pattern suggests visual behavior that underscores the importance of focusing on the patient’s story. We interpret the preference for looking more at the patient’s mouth than their eyes as an effort to understand the patient’s narrative. This approach, often referred to as lip reading or speech reading, is not only deployed exclusively for those individuals with hearing impairments but is also a natural communication strategy in general [35]. Previous studies have demonstrated that speech perception improves when combining auditory input with visual speech reading instead of relying solely on auditory cues [36].

However, it is important to carefully consider the interpretation that increased visual attention to the mouth area is the optimal strategy across all contexts. Research has shown that the eye area is used most effectively for facial identification [37], while both the eye and mouth areas are essential for recognizing emotions such as sadness, fear, and happiness [38]. Additionally, our findings align with studies indicating that the use of surgical masks impairs recognition of emotion significantly, emphasizing the critical role of the mouth in communication [39]. Synthesis of these various research findings suggests that focusing on the mouth may offer vital cues for effective communication beyond recognition tasks. Therefore, the significance of different facial areas can vary depending on the context, and further research is necessary to deepen our understanding of the relationship between facial emotion recognition and human interaction.

Additionally, the high score that the practitioner received—4.83 out of 5—on the third item of the CARE tool, which asks whether “the practitioner listened attentively to me,” suggests that the practitioner paid significant attention to reading her patients’ lips to enhance her active listening skills. The interview data further reinforced the finding that patients value empathetic behavior, particularly active listening, as a critical component of clinical empathy. Nonetheless, we need further research to explore the nuances of practitioners’ listening behaviors and attitudes in greater depth.

Lastly, it is important to consider the cultural characteristics of East Asia as a contextual backdrop to the observed behaviors. For instance, scholars acknowledge eye contact as a key component in social cognitive processes across different cultures [40]. For example, while Western cultures often perceive avoiding eye contact negatively, it is not necessarily so in Eastern cultures. Research conducted by Senju et al [41] has identified differences in the duration of gaze fixation on the eyes and mouth based on cultural background. In East Asian cultures, nonverbal communication and the subtleties of empathetic engagement may differ significantly from Western norms, thereby potentially affecting how medical professionals engage with patients. Understanding these cultural dimensions is, therefore, crucial in interpreting the implications of gaze patterns and empathetic listening in clinical settings. Further exploration of these cultural specifics could provide insights into observed visual attention behaviors and their impact on practitioner-patient interactions.

This study has several limitations. First, the eye-tracking data analyzed in this research were derived from interactions between 1 practitioner and 6 patients. While we considered the sample size adequate to confirm the feasibility of using eye-tracking technology to explore empathetic interactions in a medical environment, the small dataset may limit generalizability. Therefore, a careful interpretation of the results is required, and larger-scale studies are needed. Second, the study participants were recruited from a single practitioner’s office, which may not fully represent diverse clinical settings. Future research should investigate and analyze empathetic interactions across various clinical environments (eg, internal medicine or surgery). Third, we analyzed only the practitioner’s gaze data during practitioner-patient interactions. To gain a deeper understanding of empathy in clinical settings, future studies should explore both practitioners’ and patients’ gaze behaviors simultaneously. Additionally, conducting more thorough eye-tracking research, including research into key gaze behaviors such as fixations, saccades, and effects on pupil diameter, is necessary to expand the applicability of eye-tracking technology in clinical empathy studies.

### Conclusions

This proof-of-concept study demonstrates the potential to enhance our understanding of clinical empathy beyond the traditional reliance on self-reported data. By integrating triangulated data sources, including quantitative eye-tracking, the JSE-HP, the CARE measure, and qualitative feedback from patients on their perceptions of clinical empathy, we underscore the multifaceted nature of clinical empathy, which includes task-oriented and socially oriented dimensions. Our findings suggest that future research on clinical empathy, whether theoretical or practical (eg, empathy development training for practitioners), should address these complex characteristics. Consequently, this study advocates a dual-focused approach in the education and training of medical practitioners, emphasizing the importance of developing both theoretical and practical aspects of empathy.

## Abbreviations

AOI: area of interest
CARE: Consultation and Relational Empathy
JSE-HP: Jefferson Scale of Empathy–Health Professionals
